# Protolytic decomposition of n-octane on graphite at near room temperature

**DOI:** 10.1038/srep28493

**Published:** 2016-06-23

**Authors:** Yasushi Kawashima, Mitsumasa Iwamoto

**Affiliations:** 1 Department of Precision Engineering, School of Engineering, Tokai University, Hiratsuka, Kanagawa 259-1292, Japan; 2 Department of Physical Electronics, Tokyo Institute of Technology, 2-12-1 S3-33 O-okayama, Meguro-ku Tokyo 152-8552, Japan

## Abstract

Graphite basal surface is inert, and decomposition of n-alkanes on the graphite surface has not been discovered. We here report the evidence of decomposition of n-octanes on highly oriented pyrolytic graphite (HOPG) surface, heat-treated up to 1200 °C under high vacuum (10^−7^ Pa), at near room temperatures. Using a temperature programmed desorption apparatus equipped with a quadrupole mass spectrometer showed the production of hydrogen molecules, methane, and ethane, suggesting that the protonation of n-octane takes place on graphite surface at near room temperature. It is known that acidic functional groups are terminated at edges on the air-cleaved HOPG surface and they increase their acidity via reactions with water. However, it is most unlikely that they protonate n-alkanes at near room temperature such as superacids. We anticipate that superacidic protons, which can protonate n-octanes, are produced on the graphite surface through a novel reaction mechanism.

Graphite is a very popular material and widely used in science and engineering. The structure is simple, but its periodic flat-layer structure is serving as a source of variety material properties such as mechanical, chemical, electrical, and physico-chemical properties. Graphite is comprised of only carbon atoms, where each carbon atom is sp^2^ hybridized and creates flat structure (plane) with delocalized *π* electrons. In the graphite planes, each carbon atoms are linked to the nearest three carbon atoms with sp^2^ covalent bond (the nearest distance between carbon atoms in the plane: 1.42 Å) to form honeycomb structure. The extended structure of graphite is a consequence of the 2 p_z_ orbitals, which bond weakly between planes of the sp^2^ structures. Distance between the nearest two planes is 3.34 Å. However, we cannot obtain large single crystal graphite which enables us to determine the exact structure of graphite by using x-ray diffraction^1^,^2^ and to investigate the chemistry of graphite surface. Highly oriented pyrolytic graphite (HOPG) is usually used as a graphite sample. HOPG is composed of grains of micrometer size. The grain sizes range from a few micrometer to about 100 *μ*m. The step edges are formed on the cleaved HOPG surface. The grain boundaries are known to have somewhat lower chemical reactivity compared to the step edges^3^. It is thus believed that the chemical activity of graphite surface originates from the edges, not from the basal surface^4^,^5^.

Graphite basal plane surface is chemically inert. Carbon atoms at edge sites on graphite surface are terminated by oxygen- and hydrogen-functional groups in air^4^,^6^, and decomposition of n-alkanes due to the chemical reaction with graphite surface has not been discovered. Beyond that, alkanes are chemically very inert, and either high temperature and high pressure or an extremely strong acid is required for decomposing them, e.g., liquid phase-vapor phase thermal cracking is carried out at a high pressure up to 70 atmospheres at a moderate temperature of 350 °C to 500 °C^7^, and the cracking of alkanes is performed at a temperature of about 500 °C by bringing alkanes into contact with the catalyst such as zeolites^8^. It seems thus quite a task to find a trace of reaction for decomposing alkanes on graphite surface at near room temperatures. Therefore, the structure, composition, and behavior of alkanes adsorbed on the graphite basal surface have been extensively studied using TPD (temperature programmed desorption) and STM (scanning tunneling microscopy)^9^,^10^,^11^,^12^,^13^,^14^,^15^,^16^,^17^,^18^, under assumption that a chemical reaction never occur between alkanes and graphite. Adsorption of n-alkanes (H(CH_2_)_n_H, with n = 5 to 60) was performed on the graphite surface which is held at ~120 K^9^,^10^,^11^. The TPD measurement using quadruple mass spectrometer (QMS) showed that the desorption temperature of n-octane (melting point: 216.4 K) monolayer is about 220 K, and the desorption temperatures of n-alkanes (C_5_H_12_-C_12_H_26_) were also close to their melting points (143–263 K)^9^. Noteworthy, the results of TPD–QMS measurements showed that the thermal desorption of adsorbed n-alkanes on graphite surface occurs without any detectable decomposition or chemical reactions^9^, meanwhile the desorption of the adsorbed n-alkanes from the graphite surface occurs in a narrow temperature range near their melting points, considerably lower than room temperature. STM has been employed to study the form of n-alkanes adsorbed on HOPG surface, but no STM image has been reported on the alkane molecules shorter than n-C_16_H_34_ adsorbed on the HOPG surface at room temperature^16^. The lack of molecular resolution in STM images has been attributed to the higher surface mobility of smaller molecules^17^. Noteworthy, it is known that oxygen- and hydrogen-containing functional groups exist on the air-cleaved HOPG surface^6^, and these functional groups serve to hinder alkanes to be adsorbed on the graphite surface. If we can remove these functional groups from HOPG surface, and can bring alkanes into contact with the surface, we will have a chance to see intrinsic chemical reaction between the alkanes and the graphite basal surface, if chemical reaction is actually activated on the basal surface.

We here report a novel chemical reaction discovered between n-octane and graphite basal surface that is well heat-treated in high vacuum, by using the TPD method. The TPD measurements evidently showed that n-octane is decomposed on the graphite surface at near room temperatures and the decomposition of n-octane is accompanied by the evolution of hydrogen molecules, methane, and ethane, suggesting that protonation of n-octanes takes place on the basal graphite surface^19^,^20^. It is impossible to understand these experimental results on the basis of our accepted and conventional ideas on graphite, i.e., “graphite basal plane surface is very inert”, and the results suggests that the graphite basal surface has inherently a potentiality to activate chemical reaction leading to decomposition of n-alkanes.

## Results and Discussion

### TPD spectra of HOPG surface brought into contact with n-octanes

**Figure 1:**
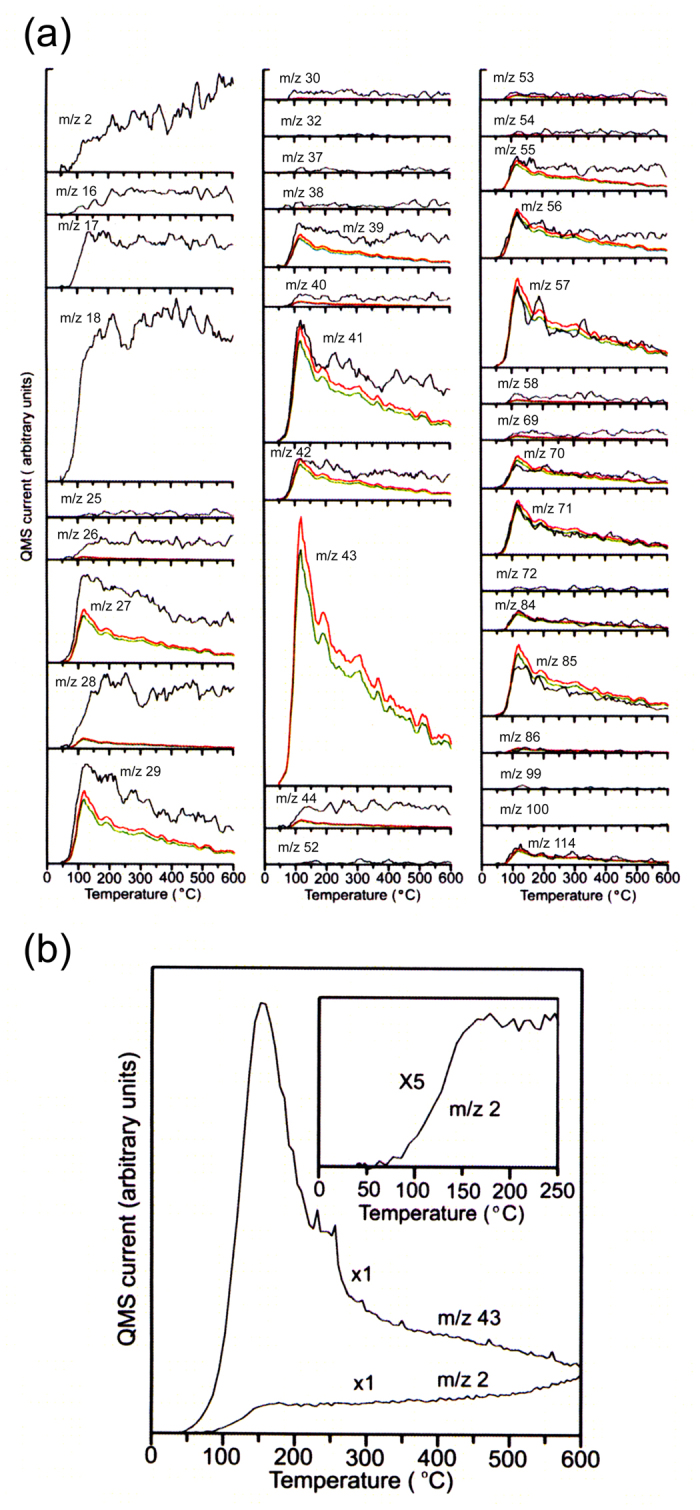
TPD spectra obtained for the HOPG sample onto which n-octane was dropped. (**a**) TPD spectra of m/z 2, 16–18, 25–30, 32, 37–44, 52–58, 69–72, 84–86, 99, 100 and 114 fragments measured for the sample in full scan mode. (**b**) TPD spectra of m/z = 2 (H_2_) and 43 measured for the sample in selected ion monitoring (SIM) mode. Black curves were obtained by subtracting the background spectra from the spectra of the HOPG sample measured immediately after dropping n-octane. Red curves show TPD curves of n-octane which are calculated based on NIST mass spectrum data of n-octane, assuming that all of the observed m/z 43 signal can be assigned to n-octane. Green curves show TPD spectra of n-octane which are calculated from the mass spectrum of n-octane in NIST database assuming that 88% of the m/z 43 signal is attributed to n-octane.

Figure 1a shows TPD spectra of m/z = 2–120 obtained for the HOPG sample at temperatures between 30 °C and 600 °C with a heating rate of 100 °C/min, where x-axis represents the temperature measured using a thermocouple placed in the sample stage of quartz. Figure 1b shows the TPD spectra of m/z = 2 and 43 measured in selected ion monitoring (SIM) mode at a heating rate of 240 °C/min. The mass spectrum of n-octane MW (molecular weight) = 114 shows the base peak at m/z 43. The observed TPD curve of m/z 43 shows temperature dependence of n-octane desorption, whereas the m/z = 2 signal indicates the generation of hydrogen molecules. The m/z = 43 ion signal starts to rapidly increase at 43 °C, while the m/z = 2 ion signal increases from ~50 °C (Fig. 1b, inset). Figure 1b evidently shows that the dehydrogenation reaction is activated on the graphite surface.

**Figure 2:**
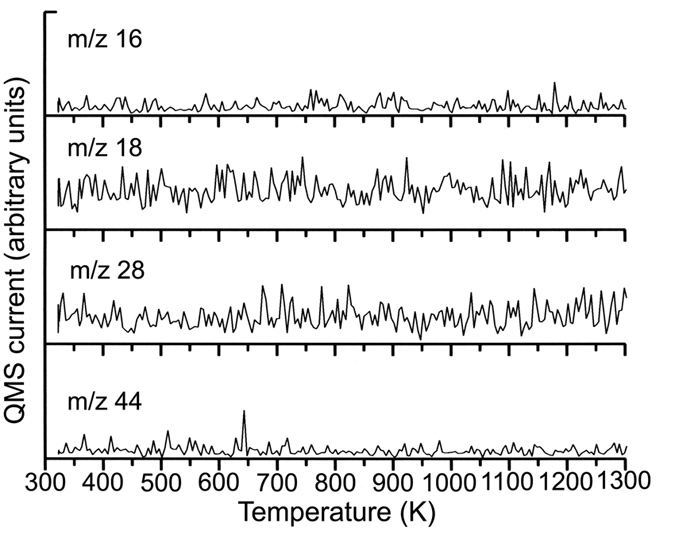
TPD curves of m/z = 16, 18, 28 and 44 signals of HOPG sample heated up to 1200 °C under ultrahigh vacuum (~10^−7^ Pa) which were measured without taking the sample out of the ultrahigh vacuum chamber.

TPD spectra of HOPG sample heated up to 1200 °C under ultrahigh vacuum (~10^−7^ Pa) were measured without taking the sample out of the ultrahigh vacuum chamber. The TPD curves for m/z = 16, 18, 28 and 44 signals up to 1300 K are shown in Fig. 2. The m/z = 16, 18, 28 and 44 signals are observed when oxygen- and hydrogen-functional groups desorb from the HOPG surface^6^. Oxygen- and hydrogen-containing functional groups on air-cleaved HOPG can be removed by thermal treatment^6^. It has been demonstrated that all the functional groups on the HOPG surface can be removed by heat treatment above 1273 K^6^. Figure 2 does not show the m/z = 16, 18, 28 and 44 signals at temperatures up to 1273 K. Therefore, it is confirmed that all functional groups on our HOPG sample are removed by heat treatment up to 1200 °C.

### Decomposition of n-octane on HOPG surface

On the basis of NIST(The National Institute of Standards and Technology) database of mass spectra on straight chain and branched alkanes, alkenes, and their isomers with 1–8 carbon atoms, we confirmed that a part of n-octane dropped onto the graphite surface decomposes into smaller alkanes or alkenes with 1–3 carbon atoms as follows; Assuming that all of the m/z 43 ion signal are attributable to n-octane, TPD curves of n-octane were calculated from the m/z 43 TPD curve using NIST mass spectrum data, and the results were displayed by red curves in Fig. 1a. The calculated signal intensities of m/z 57, m/z 70, m/z 71 and m/z 85 (red curves) are larger than those (black curves) of the measured ones, respectively. This fact clearly shows that a part of the source of the measured m/z 43 ion signal is not the n-octane. TPD spectra of n-octane calculated from the mass spectrum of n-octane in NIST database assuming that 88% of the m/z 43 ion signal attributes to n-octane are plotted by green curves in Fig. 1a. These plots show that green curves roughly agree with the experimental observed curves (black curves) in the m/z 57, m/z 70, m/z 71 and m/z 85 TPD spectra, indicating that the green curves are TPD curves of n-octane.

**Figure 3:**
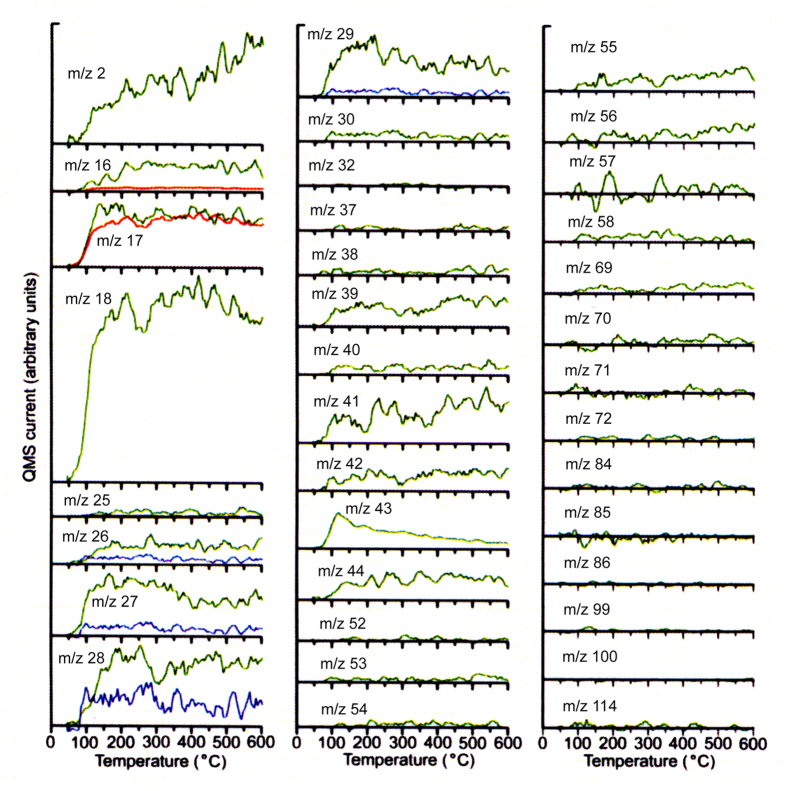
TPD spectra of m/z 2, 16–18, 25–30, 32, 37–44, 52–58, 69–72, 84–86, 99 and 100 fragments that do not originate in n-octane. The TPD spectra were obtained by subtracting the TPD spectra of n-octane shown by green curves from the observed TPD spectra (black curves) in Fig. 1a. In TPD spectra of m/z 16, 17, red curves show TPD spectra due to the water fragment ions OH^+^ (m/z 17) and O^+^ (m/z 16) which are calculated from the mass spectrum of water in NIST database and TPD curve of m/z 18 (water). In TPD spectra of m/z 26, 27, 28 and 29, blue curves show TPD spectra of the ethane fragment ions (m/z 26, 27, 28 and 29) that are calculated based on the mass spectrum of ethane in NIST database and TPD curve of m/z 30 (ethane).

Figure 3 shows TPD spectra (green curves) obtained by subtracting the TPD spectra of n-octane shown by green curves from the observed TPD spectra (black curves) in Fig. 1a. Therefore, these TPD spectra are not due to the n-octane. Results show that with increasing temperature, the intensity of the m/z 43 signal decreases whereas those of the m/z 41 and 55 signals increase. The increase of the m/z 41 and 55 signals support the presence of unsaturated carbon compound such as alkenes and the m/z 43 signal is generally identified as alkane. These facts suggest that dehydrogenation of alkane to alkene occurs and it accompanies with evolution of hydrogen (see Fig. 1b).

As can be seen from Fig. 3, the intensities of m/z 27, 28 and 29 ion signals are much larger than those of m/z 30–114 ion signals. In the mass spectra of straight chain alkanes C_4_H_10_, C_5_H_12_, C_6_H_14_, C_7_H_16_ and C_8_H_18_ and their isomers, the intensities of m/z 27, 28 and 29 signals are smaller than that of either m/z 43, 57, 71, or 85 signal. From these facts, the intensities of m/z 27, 28 and 29 signals cannot be explained by assuming the mass spectra of C_4_-C_8_ n-alkanes and their isomers.

Similarly, in the mass spectrum of propene C_3_H_6_, the intensities of m/z 27, 28 and 29 signals are smaller than that of m/z 42 signal. In the mass spectra of 1-butene C_4_H_8_ and its isomers, the intensities of m/z 27, 28 and 29 signals are smaller than that of m/z 56 signal. In the mass spectra of 1-pentene C_5_H_10_ and its isomers, the intensities of m/z 27, 28 and 29 signals are less than or almost equal to that of m/z 70 signal. In the mass spectra of 3-hexene C_6_H_12_ and its isomers, the intensities of m/z 27, 28 and 29 signals are smaller than that of either m/z 55, 56, or 69 signal. In the mass spectra of 1-heptene C_7_H_14_ and its isomers, the intensities of m/z 27, 28 and 29 signals are also smaller than that of either m/z 55, 56, or 69 signal. In the mass spectra of 1-octene C_8_H_16_ and its isomers, the intensities of m/z 27, 28 and 29 signals are less than that of m/z 55 signal. The relative intensities of m/z = 27, 28, 29, 41, 42, 43, 55, 56, 69 and 70 signals obtained from the NIST mass spectrum database of propene, 1-butene, 1-pentene, 3-hexene, 1-heptene, and 1-octene are shown in Table 1, where the intensities of the base peaks of these alkenes are assigned an abundance of 100.

From these facts mentioned above, it is concluded that most of the m/z 27, 28 and 29 signals are not due to fragments from C_3_-C_8_ alkenes.

Figure 3 shows that the intensities of m/z 27, 28 and 29 ion signals are considerably larger than those of m/z 43, 55, 56, 57, 69, 70, 71, 84 and 85, suggesting that butane, ethane, methane, and ethylene, which have the number of carbon atoms smaller than or equal to 3, were produced on the graphite surface. That is, n-octane decomposed into C_1_-C_3_ alkanes or C_2_ alkene.

### Generation of ethane

Figure 3 demonstrates that the ion m/z 30 was constantly produced at a temperature from about 70 °C. With the exception of ethane, the mass spectra of alkanes, alkene, and their isomers, which have a chance of being generated by decomposition of n-octane, do not show a peak at m/z = 30 or even if they show it, it is very small compared to their base peaks. Therefore, the non-zero TPD spectrum of the m/z = 30 suggests the generation of ethane. The TPD curves for m/z = 25, 26, 27, 28 and 29 signals due to the ethane were calculated on the basis of the TPD curve of the m/z = 30 and the mass spectrum of ethane (NIST database), which are plotted by blue curves in Fig. 3. Results account for the generation of ethane, and all the fragment ion peaks in the mass spectrum of ethane are observed.

### Generation of methane

**Figure 4:**
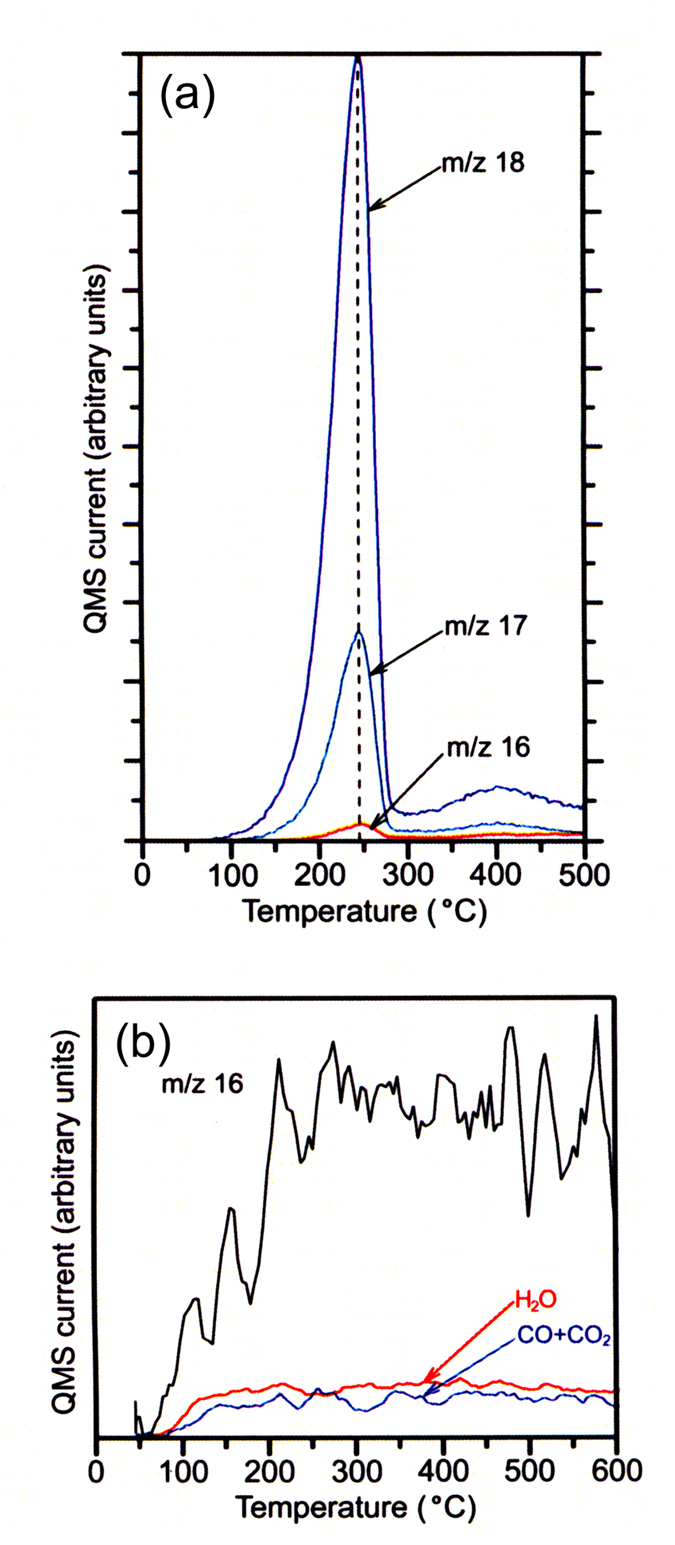
TPD spectra of water and m/z 16 ion signal from the HOPG sample. (**a**) TPD spectra of the m/z 16, 17 and 18 of water evolved by thermal decomposition of calcium oxalate monohydrate (CaC_2_O_4_ · H_2_O) which were measured by the TPD apparatus used in the experiments. (**b**) TPD spectrum (black curve) of m/z 16 ion signal obtained for the HOPG sample onto which n-octane was dropped.

Figure 4a,b show TPD spectra of calcium oxalate monohydrate (CaC_2_O_4_ · H_2_O) which evolves only water at ~245 °C and the TPD spectrum of the m/z = 16 observed for the HOPG samples, respectively. According to the TPD spectra in Fig. 4a, it is found that in mass spectrum of water measured by the used apparatus, ion (OH^+^) at m/z 17 has a relative intensity of 26.5% in comparison to that of the base peak at m/z 18 and ion (O^+^) at m/z 16 has a relative intensity of 2.3%. TPD curves of m/z = 17 and m/z = 16 calculated from the TPD curve of H_2_O (m/z = 18) on the basis of the TPD spectra of CaC_2_O_4_ · H_2_O are plotted by red curves in Fig. 3. Although the calculated TPD curve of m/z = 17 agrees with the observed TPD spectrum of m/z 17, the intensity of m/z 16 ion signal calculated from the TPD curve of H_2_O is considerably smaller than the measured intensity of m/z 16 signal. TPD curve of m/z = 16 (O^+^) originating in water is plotted by red curve (see TPD spectrum of m/z = 16 in Fig. 3). Further, TPD spectrum of m/z = 32 in Fig. 3 shows that no oxygen molecule (O_2_) was detected at temperatures between 30 and 600 °C. Therefore, the observed m/z 16 ion signal does not arise from oxygen molecule. According to the NIST database, with the exception of methane, mass spectra of hydrocarbons do not show peak at m/z = 16, even if they show the peak at m/z = 16, it is extremely small compared to their base peaks. Assuming that in Fig. 3, all the m/z 28 and 44 signals are due to carbon monoxide (CO) and carbon dioxide (CO_2_), respectively, the sum of m/z 16 ion signals that come from CO and CO_2_ molecules was obtained at temperatures between 30 and 600 °C from the observed TPD curves of m/z = 28 and m/z = 44 on the basis of NIST database. The sum of TPD curves of the m/z = 16 signals due to CO and CO_2_ is plotted by a blue curve in Fig. 4b. TPD curve of m/z = 16 (O^+^) originating in water, which is calculated from the TPD curve of H_2_O (m/z = 18) on the basis of the TPD spectra of CaC_2_O_4_ · H_2_O, is also shown by red curve in Fig. 4b. Most of the observed m/z 16 ion signal arises from methane, not from oxygen. Thus the production of methane is confirmed.

### Protonation of n-octane

It is known that alkanes thermally decompose at temperatures in the region 500–800 °C^21^, due to thermal cracking, where free radicals are serving as active intermediates. On the other hand, our TPD measurements showed that decomposition of n-octane to C_1_-C_3_ hydrocarbon and dehydrogenation of alkanes to alkenes occur on the graphite surface at a temperature from about 70 °C. This is not due to thermal cracking, but possibly due to catalytic one. The TPD experiments showed that dihydrogen, methane, and ethane are produced in the cracking. This result indicated that this decomposition is not based on the classical (bimolecular) mechanism of catalytic cracking including β-scission and hydride transfer where the smallest alkane cracking product is propane^19^,^20^.

Here we propose that the protolytic cracking on the graphite surface^19^,^20^ is the most possible mechanism that accounts for the results of our TPD measurements. The production of methane and ethane suggests that five-coordinate alkanium ions having three center two-electron bonds (carbonium ions)^22^ are formed via protonation of alkanes by an extremely strong acid (e. g., a superacid) that has very high proton donor strength and the alkanium cations decompose to produce hydrogen (H_2_) or an alkane, for example, methane, ethane, and a carbenium ion.

In our experiment, HOPG sheets are heated up to 1200 °C under ultrahigh vacuum conditions (~10^−7^ Pa), to remove all contaminates such as oxygen- and hydrogen-containing functional groups on the air-cleaved HOPG surface. However, carbon atoms at the edge will be terminated by hydrogen and oxygen functional groups^4^,^6^ before n-octane being dropped on the HOPG samples in air (see Methods). Nevertheless, on the surface of heat-treated HOPG sheets almost all the adsorption sites are chemically accessible for n-octane molecules. Since the step edges of graphite are hydrophilic and possess a number of functional groups (phenolic, carboxylic, and ketonic groups)^23^,^24^, the hydrophilic edges are capable of strongly adsorbing water vapors in air. These oxygen functional groups that terminate the edges of graphite have acidic properties and increase their acidities via reactions with water^25^. However, according to a rough estimate of the acidic properties of graphene oxide (GO)^25^, it is clear that their acidities (pK_a_) are orders of magnitude weaker than those of superacids that can protonate n-alkanes at near room temperature. We therefore argue that superaidic protons, which serve to protonate n-octane, are generated not at the edge sites and that the protonation of n-octane occurs on graphite basal plane surface (ref. 26). The observed phenomenon shows that superacidic naked protons, which can protonate n-octane, are generated on the graphite surface brought into contact with n-octane. A novel idea will be required to account for the generation of superacidic protons on the graphite surface. We anticipate that the superacidic protons move freely without activation energy on the graphite surface. The superacidic protons can give rise to room temperature superconductivity^27^.

## Methods

### TPD apparatus

The chemical reaction between graphite and n-octane was studied under ultrahigh vacuum conditions (~10^−7^ Pa) by using TPD apparatus (ESCO. Ltd.) equipped with a quadrupole mass spectrometer (QMS) (QMS system, QMG 422, quadrupole analyzer QMA 125: Pfeiffer Vacuum GmbH) in two operational modes, i.e., full scan and selected ion monitoring (SIM) modes^28^. In the TPD apparatus, a sample is placed on the sample stage of quartz and an infrared light is employed for the heating. The infrared light passing through the quartz stage uniformly heats the sample. The temperature of the sample is monitored by a thermocouple placed between the sample and the quartz stage. The sample temperature is controlled using the temperatures monitored by the thermocouple.

### Samples

HOPG sheets were purchased from Momentive Performance Materials Inc. and their grade was ZYA. N-octane was purchased from Alfa Aesar, A Johnson Matthey Company (purity <98%). Four HOPG sheets (5 × 12 × 0.36 (average) mm) were prepared from the each purchased sheet and both sides of these four sheets were cleaved with scotch tape in air before experiment.

### TPD measurements

It has been known that oxygen- and hydrogen-containing functional groups exist on the air-cleaved HOPG surface^6^, and these functional groups hinder the adsorption of alkanes on the graphite surface. In order to remove all these functional groups, the prepared HOPG sheets were heated up to 1200 °C under ultrahigh vacuum (~10^−7^ Pa). After that, these sheets were cooled to room temperature. As soon as the sheets were taken out from the ultrahigh-vacuum chamber, a 30 *μ*l solution of n-octane was dropped onto the surface in air, using a micropipette in less than 15 s. These HOPG sheets were samples used in this experiment. After the completion of this procedure, the HOPG samples were inserted into the ultrahigh-vacuum chamber for TPD measurements. TPD measurements were carried out at temperatures from room temperature to 600 °C with a heating rate of 100 K min^−1^ under ultrahigh vacuum (~10^−7^ Pa). The mass spectrometer operated in the electron impact ionization mode at 70 eV. In the full scan mode (m/z 1–120), electron impact ionization (EI) mass spectra were recorded at 70 eV electron energy. Before each measurement, the background spectrum was recorded using as heat-treated HOPG sheets, from which no peaks were observed in the range of m/z 1–120 up to 800 °C.

## Additional Information

**How to cite this article**: Kawashima, Y. and Iwamoto, M. Protolytic decomposition of n-octane on graphite at near room temperature. *Sci. Rep.* **6**, 28493; doi: 10.1038/srep28493 (2016).

## Figures and Tables

**Table 1:** Relative intensities of m/z = 27, 28, 29, 41, 42, 43, 55, 56, 69 and 70 ion signals of propene, 1-butene, 1-pentene, 3-hexene, 1-heptene, and 1-octene.

	m/z 27	m/z 28	m/z 29	m/z 41	m/z 42	m/z 43	m/z 55	m/z 56	m/z 69	m/z 70
propene	38.7	1.4	0	100	70.4	2.3	/	/	/	/
1-butene	25.1	28	12.5	100	3.3	0.1	18.1	38.8	/	/
1-pentene	18.3	2.4	21	43.9	100	4.1	65.3	2.9	1.9	39.5
3-hexene	19.6	1.6	18.8	66.5	61.1	14.5	100	26.6	26.7	1.5
1-heptene	25.7	4.9	55.9	96.8	54.9	15.9	67.6	100	31.1	44.2
1-octene	25.1	5.2	35.4	81.8	66.1	100	99.2	86.6	44.4	85.7

These were calculated from NIST mass spectrum database. The intensities of the base peaks of these alkenes are assigned an abundance of 100.
